# Towards the personalization of gelatin-based 3D patches: a tunable porous carrier for topical applications

**DOI:** 10.1007/s13346-023-01294-y

**Published:** 2023-01-12

**Authors:** Ricardo Ribeiro, Sara Bom, Ana M. Martins, Helena M. Ribeiro, Catarina Santos, Joana Marto

**Affiliations:** 1 Research Institute for Medicines (iMed.ULisboa), Faculty of Pharmacy, Universidade de Lisboa, Lisbon, 1649-003 Portugal; 2 CQE, Instituto Superior Técnico, Universidade de Lisboa, Av. Rovisco Pais, Lisbon, 1049-001 Portugal; 3 EST Setúbal, Instituto Politécnico de Setúbal, CDP2T Setúbal, Portugal

**Keywords:** 3D (bio)printing, Hydrogel, Personalized therapies, Topical delivery systems

## Abstract

Cell-free based therapies, for example, the use of the cell secretome, have emerged as a promising alternative to conventional skin therapies using bioactive and, when combined with 3D printing technologies, allow the development of personalized dosage forms. This research work aimed to develop gelatin-based patches with controlled network topology via extrusion 3D printing, loaded with cell culture medium as a model of the secretome, and applicable as vehicles for topical delivery. Inks were optimized through rheological and printing assays, and the incorporation of medium had minor effects in printability. Regarding network topology, grid infills rendered more defined structures than the triangular layout, depicting clearer pores and pore area consistency. Release studies showed that filament spacing and infill pattern influenced the release of rhodamine B (model bioactive) and bovine serum albumin (model protein). Moreover, the grid patches (G-0.7/1/0.7), despite having around a seven-fold higher mean pore area than 0.7-mm triangular ones (T-0.7), showed a similar release profile, which can be linked to the network topology of the printed structures This work provided insight on employing (bio)printing in the production of carriers with reproducible and controlled pore area, able to incorporate cell-derived secretome and to be quickly tailored to the patient’s lesions.

**Graphical Abstract:**

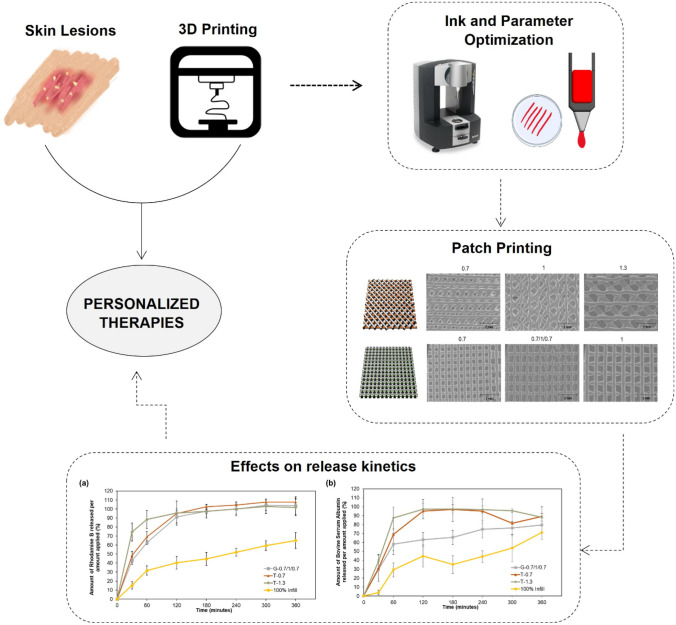

**Supplementary Information:**

The online version contains supplementary material available at 10.1007/s13346-023-01294-y.

## Introduction

Skin diseases and lesions greatly impact the patients’ quality of life, justifying research efforts to find successful treatments. Regarding skin diseases, in particular inflammatory chronic diseases such as psoriasis or atopic dermatitis, which often require immunosuppressive treatments, cellular-based therapies may provide a promising alternative therapy to conventional molecules [1]. These include cell-free approaches which rely on the pool of secreted molecules by a certain type of cells, the secretome, which is the scope of this work. Current conventional treatments for such diseases usually consist in the topical administration of conventional pharmaceuticals, such as glucocorticoids and vitamin D analogs, or in the oral administration of cyclosporins and retinoids [1, 2]. Although generally less effective than oral or parenteral drug administration due to the different mechanisms of skin absorption, topical treatments represent a very attractive method to battle skin diseases. In chronic inflammations, immunosuppressive drugs are typically administered systemically, having a long-term effect, but acting globally. Thus, the use of immunosuppressive drugs which act locally is preferable in these types of skin lesions [2].

Despite the efforts made to develop effective drugs for the treatment of inflammatory skin diseases, the cost and the non-curative profile of the current therapeutic systems highlight an urgent need to develop novel medicines and/or pharmaceutical forms [2, 3]. Naturally, the immunogenic profile of such diseases makes them a common target of therapies that resort to mesenchymal stem cells (MSCs) and their immunomodulatory activity, a rationale that already showed promising results in both animal and human models [4–6]. However, some stem cell types, including MSCs, carry tumorigenic risks, besides requiring a costly and extensive ex vivo expansion that may lead to cellular senescence and its associated mutations, further enhancing the associated risks [7, 8]. This dynamic has driven research to cell-free therapies, which rely on the paracrine effects of molecules secreted by immunomodulatory cells, instead of using the cells themselves. Furthermore, secreted bioactives (e.g., conditioned media, secretome-derived exosomes) just like conventional pharmaceuticals, can be loaded into 3D-printed vehicles which can be customized to the patient in both design and dosage form [9]. This is an advantageous methodology due to safety, manufacturing, handling, and storage purposes, besides overcoming limitations inherent to cell printing approaches, which are more laborious and must address the need to create a suitable environment for cell viability and growth [7]. However, to be topically administered, the vehicles carrying cellular-based bioactives need to be developed with suitable materials, which can release the cargo in a controlled way [3]. This rationale led to the development of soft materials and hydrogels, capable of harboring important biological factors, as potential vehicles for topical administration, which is the subject of this research study [10, 11]. Hydrogels are 3D swollen polymeric matrices with high water content that do not solubilize, thus resembling soft tissues, particularly the extracellular matrix (ECM) [10, 11]. These structures are biocompatible and capable of encapsulating bioactive products in a friendly environment and with a uniform spatial distribution. Another advantage of utilizing semi-solid hydrogels is that these materials, when used for topical delivery via wound dressings, have enough flexibility to arrange themselves according to the contour of the body and can also provide a moist environment that accelerates wound healing [12, 13]. Furthermore, the possibility to produce hydrogel vehicles resorting to 3D (bio)printing is opening a new prospect to the development of innovative, cost-effective, personalized, and individualized treatment platforms [10, 13, 14]. Alongside, 3D technology can be a solution to repurposing the current therapeutic systems available on the market by modulating the release through advanced printing strategies, including the possibility to create medical devices which can be customized to the patient in features such as pore size, shape, mechanical endurance, and drug release profile [3, 15]. However, the materials used in 3D (bio)printing and the design features of the constructs, such as number of layers, layer orientation, filament spacing, and pore area (PA), can have a major influence in their applicability [10, 16]. Thus, mapping the relationships between such parameters is crucial to achieve a fast customization, since the uniqueness of lesions in skin diseases implies that each patient has different design requirements and restrictions. Teoh et al. [12] addressed this problem by characterizing the release profile of a pool of chitosan methacrylate wound dressings with a variable number of layers. The authors observed that a larger number of layers favored a prolonged release and tested several combinations of levofloxacin-loaded and plain hydrogel layers to further tailor the release profile. Tytgat et al. [17] used a grid design to analyze the effects of varying filament spacing on methacrylate gelatin scaffolds in their compressive modulus. The results showed that a larger filament spacing led to dressings which were mechanically inferior to those with a shorter distance between adjacent filaments. Milojevic et al. [13] highlighted that highly porous matrices are beneficial as wound dressings because they promote moisture retention, facilitate the transport of nutrients and oxygen, and allow the ingrowth of underlying tissue. However, this study also emphasized that a larger macroporosity reduced the mechanical performance of the patch. Overall, these studies emphasize the importance of understanding how to modulate the porosity level of the hydrogels’ patches by varying the printing settings.

Nevertheless, 3D printing applications still present some major obstacles which must be addressed before the technology can be successfully implemented in skincare treatments worldwide. There are three main stages in 3D printing—pre-printing, printing, and post-printing, each having distinct interrelated factors affecting the quality of the printed structure [18, 19]. As previously highlighted by our group [20], a current major drawback is the lack of standardization in nomenclature and methods, which leads to a notorious lack of inter-experimental reproducibility. The optimization of printing parameters is pivotal to achieve high-quality constructs, but this phase is often performed in a unique approach in each study [21, 22]. Consequently, this poor reproducibility impairs comparisons across literature and hinder the development of mainstream personalized therapies [18, 19]. To conclude, the potential adoption of personalized therapies will depend on the level of singularization that can be proposed to the patient. Therefore, to achieve a high degree of customization that better suits the patient’s needs, it would be ideal to individualize not only the design at the computer-aided design (CAD) model level, but also the parameters related to the design, such as the tensile strength or the pore area and associated release rate. Thus, it is essential to understand the intricacies of altering these design’s parameters and build a basis of guidelines for the fast printing of patches with desired fundamental features, with high reproducibility.

This research work aimed to use different design features to develop gelatin-based 3D extrusion-printed hydrogel patches with variable network topologies, loaded with cell culture medium (CCM) as a representative model of a pool of secretome-derived molecules, for the topical delivery of cell-free bioactives for skin applications, and to study their rheological performance and release kinetics. To achieve such goal, optimization and characterization methodologies which are common in extrusion-based bioprinting were used, thus allowing to customize printing parameters to the ink in use and, consequently, to obtain the best printing accuracy possible. Additionally, the release kinetics and underlying mechanisms were also studied, with the goal of profiling the release for each type of printed patch. This work contributes to the future achievement of a fast and practical set of guidelines which can provide a personalized carrier with unique pore area, infill pattern, and release profile, prompted to be readily adjusted to the desired application, which combines the therapeutic potential of some cell types while simultaneously avoiding the inherent drawbacks of directly employing these cells in 3D (bio)printing procedures.

## Materials and methods

### Materials

Type B gelatin powder was purchased from Acofarma (Madrid, Spain). Sucrose was obtained from Fisher Scientific (Hampton, USA). Glycerin was acquired to Lacrilar (Torres Vedras, Portugal). Cell culture medium (α-MEM), fetal bovine serum (FBS), and rhodamine B (RB, purity degree ≥ 95%) were obtained from Sigma-Aldrich (USA). Albumin bovine fraction V (BSA) MB grade was purchased from NZYTech (Lisboa, Portugal). Purified water was obtained by reverse osmosis and electrodeionization (Millipore®, Elix 3), followed by filtration (filter pore 0.22 µm) and sterilization.

### Methods

#### Preparation of gelatin-based hydrogel inks

Gelatin-based hydrogel inks were prepared in a water bath (Nahita International, UK) at 50–55 °C for 1 h. Glycerin (GLY; 10%) was initially mixed with 40% CCM and gently stirred in the water bath for 5 min, until a homogeneous solution was obtained. The CCM (α-MEM) used in the formulations had previously been in contact with a culture of human umbilical cord-derived MSCs (UCX®) supplemented with 10% FBS. This mixture was then slowly blended with gelatin (GEL; 40%) and sucrose (SUC; 10%) and discontinuously stirred for 55 min, to promote an adequate crosslinking and homogenous ink—GEL40-CCM40. As a control (GEL40), a formulation without CCM was employed. For the release studies, RB and BSA were incorporated at the final stage of the hydrogel ink preparation, at 1 mg/mL and, after printing, the patches were kept in the dark at room temperature (RT).

#### Pre-printing and printing optimization

A series of optimization and characterization tests were performed to adjust the printing parameters to the ink in use and, sequentially, to observe a possible influence of the CCM in printability.

##### Rheological analysis

The rheological analysis was performed with a controlled stress Kinexus Lab + rheometer (Malvern Instruments, Malvern, UK) utilizing a parallel plate geometry (upper radius = 10 mm) in a 0.5-mm gap, which aimed at mimicking the patch height. About 0.5 g of each formulation was placed in the lower geometry before lowering the upper geometry. All rheological measurements were performed at RT. Oscillatory tests were carried out to analyze viscoelastic properties at different temperatures (temperature ramp analysis). Frequency was kept at 1 Hz and the shear strain at 1%, with temperature decreasing from 50 to 25 °C at a rate of 2.5 °C/min for 10 min. The sol/gel transition temperature was determined by analyzing the crossover point between storage and loss modulus. Afterwards, to inspect the thixotropic behavior of the hydrogels, three-time interval sequences were designed to analyze the viscosity recovery behavior while mimicking the forces exerted on the ink during the printing process. The first stage simulates the resting period inside the cartridge prior to extrusion; therefore, the formulation was kept at 42 °C while a constant shear rate of 0.1 s^−1^ was exerted for 6 min. In the second stage, also at 42 °C, the shear rate was increased to 100 s^−1^ for 7.5 min, to simulate the stresses sustained while extruding. Lastly, in the third stage, the ink experienced a cooling-down from 42 to 25 °C and the shear rate returned to 0.1 s^−1^ for 10 min, which mimics the period after deposition. The frequency was maintained at 1 Hz in all stages.

##### 3D printing process

All printing procedures were carried out on an Allevi2 bioprinter (Allevi, Philadelphia, USA). Inks were printed after a stabilization period of at least 10 min in the cartridge. The cartridge was also pre-heated to minimize temperature variations within it. After stabilization, the formulations were pneumatically extruded through a specific nozzle: 27 gauge metal tapered 0.25″ tip (inner diameter = 0.335 mm). Plastic was selected as the support material to improve adhesion during patch printing.

##### Extrudability and printing fidelity

1. Extrusion test

The filament extrusion drop test analyzed the extrudability via visual screening of the printing outcome. First, this test was used to define the ideal pressure by increasing by 5 Psi increments until the material extruded evenly. After defining the ideal pressure as 15 Psi, the test was performed for the temperature window selected in the rheological tests*,* from 39 to 48 °C, to validate the printing temperature.

2. Line test

A series of 40-mm linear filaments were printed under the temperature range previously defined (39 to 48 °C) and constant pressure (15 Psi), at printing speeds between 5 and 30 mm/s (*n* = 3 for each condition), to study how these parameters affect the filament width (*W*′) and length (*L*′) indexes.

The *W*′ is used to study the degree to which a filament spreads laterally and can be calculated with Eq. (1) [18, 23–25]:

1 $$W^{\prime}=\frac{Printed\ filament\ width}{Needle\ diameter}$$

The length of the printed filament was divided by the length of a theoretical, perfectly uniform strand, to obtain the length index (*L*′) [26]:

2 $$L^{\prime}=\frac{length\ of\ printed\ filament}{length\ of\ theoretical\ filament}$$

The filament width and length (*n* = 3) were measured resorting to ImageJ® software.

3. Flow rate

After determining the material density, the mass of the printed samples was used to establish the output volume as a function of the time spent in extrusion (10, 20, and 30 s). Flow rate (*FR*) was determined through the following equation:

3 $$FR=\frac{Volume}{Time}$$

This assay was performed at 42 °C, which was defined as the ideal printing temperature in the previous tests.

#### Printing porous multilayer patches

**Fig. 1:**
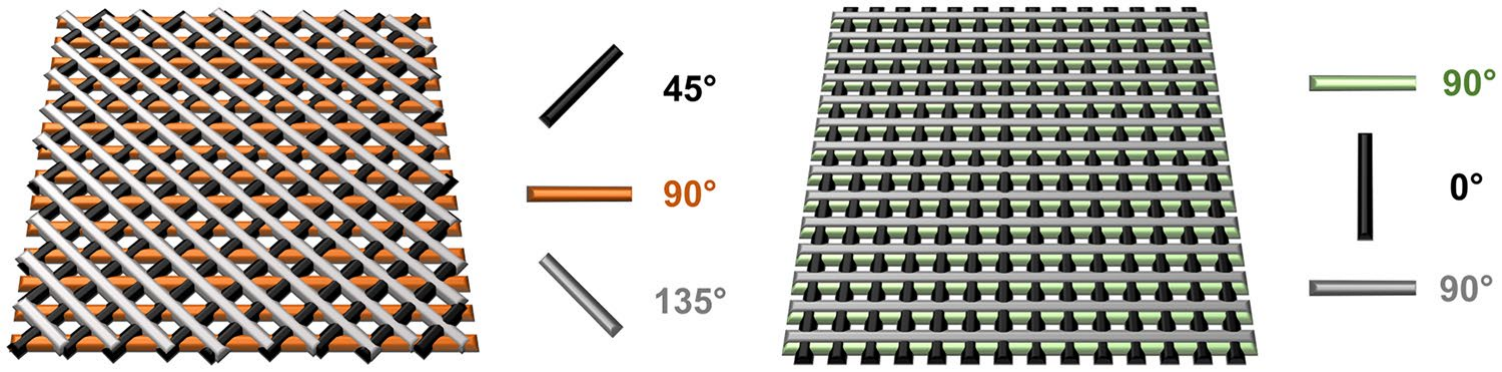
Design and layer orientation of triangular (left) and grid (right) patches

To evaluate the significance of creating adequate porosity in the 3D patches, patches with several infill patterns were printed in the previously optimized conditions. The infill pattern and its orientation and nomenclature are shown in Table 1 and Fig. 1.

**Table 1:** Infill pattern, number of layers, orientation, and nomenclature of the different printed patches

**Infill pattern**	**Layer number**	**Orientation**	**Line distance (mm)**	**Nomenclature**
Grid (G)	3	0°-90°-0°	0.7	G-0.7
			1	G-1
			0.7–1–0.7	G-0.7/1/0.7
Triangular (T)	3	45°-90°-135°	0.7	T-0.7
			1	T-1
			1.3	T-1.3

Measurements of pore area were performed using the ImageJ^®^ software

#### In vitro release studies

For the release experiments, CCM-based patches with different infills and a structure with dimensions 20 mm × 20 mm × 0.45 mm (*n* = 6, for each assay) loaded with RB (model bioactive) or BSA (model protein) were cut into smaller patches, preserving the center regions, and discarding the edges, and suspended in 1.5 mL of phosphate-buffered saline, pH 7.4 (PBS) inside microtubes, at RT and in static conditions. Samples (200 μL) were collected at specific time points, up to 6 h, and the volumes were replaced with fresh PBS at the same temperature. Between measurements, the samples were kept in the dark to protect RB from light. The absorbances of the collected samples were measured at 580 nm (maximum emission wavelength of RB [27]) and 280 nm (absorption wavelength of proteins [28]), in a Fluostar Omega microplate reader (BMG Labtech, Ortenberg, Germany). The percentage of RB and BSA released into the medium was calculated using the following equation:

4 $$\mathrm{Cumulative\ release\ percentage}=\sum_{t=0}^{t}\frac{{M}_{t}}{{M}_{0}}\times 100$$

where *M*_*t*_ is the cumulative amount of RB or BSA released at each sampling time point, *t* is time, and *M*_0_ is the initial weight of the RB or BSA in the GEL-based patches.

The release studies were performed five times, and mean values of cumulative release (%) were plotted against time. The data obtained were computed using DDsolver [29], an Excel-plugin module, and different kinetic models were fitted to the resultant data:

1. Zero-order kinetics
  $$F={K}_{0}\times t$$

where *K*_0_ is the zero-order release constant.

2. First-order kinetics
  $$F=100\times (1-{e}^{-{K}_{1}\times t})$$
  where *K*_1_ is the first-order release constant

3. Higuchi model
  $$F={K}_{H}\times {t}^{1/2}$$
  where *K*_*H*_ is the Higuchi release constant

4. Korsmeyer-Peppas model
  $$F={K}_{KP}\times {t}^{n}$$
  where *K*_*KP*_ is the release constant incorporating structural and geometric characteristics of the drug-dosage form and *n* is the diffusional exponent indicating the drug-release mechanism

5. Weibull model
  $$F=1-\mathrm{exp}\left[\frac{-(t-{{T}_{i})}^{\beta }}{\alpha }\right]$$
  where *α* defines the time scale of the process; ${T}_{i}$ represents the lag time before the onset of the dissolution or release process; and *β* is the shape parameter which characterizes the curve as either exponential (*β* = 1), sigmoid, S-shaped, with upward curvature followed by a turning point (*β* > 1), or parabolic, with a higher initial slope and after that consistent with the exponential (*β* < 1)

In all models, *F* is the fraction (%) of released drug at time *t*. The adjusted coefficient of determination (*R*^2^_adjusted_) was calculated for each model and used as an estimate of the goodness-of-fit, i.e., the model ability to describe a given dataset. The *R*^2^_adjusted_ values and the Akaike minimum information theoretical criterion (AIC) were used as a measure of fit to compare the different models. When comparing several competing models, the best fitting model was the one with maximum ${R}_{\mathrm{adjusted}}^{2}$ and minimum AIC.

## Results and discussion

### Influence of CCM incorporation on the pre-printing and printing parameters

It has been described that the use of gelatin alone as an ink in 3D printing can lead to constructs with poor mechanical properties and high degradation rates [30]. Therefore, in this study, gelatin was blended with glycerin, which is a natural humectant, that traps more water and renders more flexible structures [31], and sucrose, which is a low toxicity crosslinker that boosts a further entangled polymeric network [32] and enhances the mucoadhesive property of the hydrogel [33]. The first goal was to study the influence of using CCM (as a representative model of secretome-based therapies) on the gelatin-based formulation ink, in the rheological features and the quality of patch printing. At an initial stage, different CCM concentrations (10 to 40%) were tested (data not shown) and GEL40-CCM40 was the formulation selected to be further analyzed since it contains higher medium concentration, granting possible higher incorporation of biological materials or derivates.

#### Pre-printing analysis and validation of rheological results through extrudability experiments

**Fig. 2:**
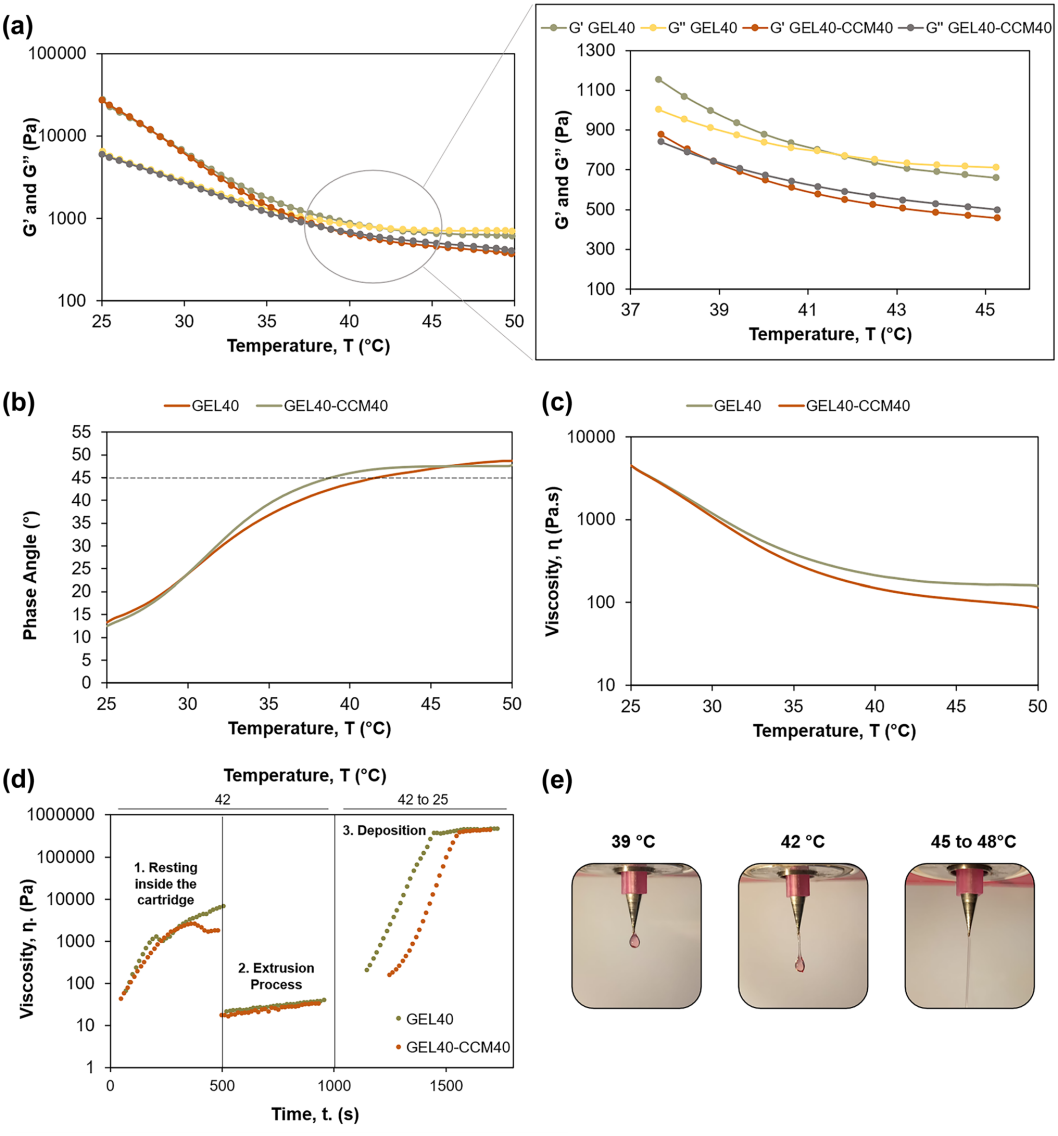
Rheological pre-printing analysis and validation of results through extrudability. **a** Loss and elastic modulus variation with temperature, **b** phase angle variation with temperature, **c** viscosity variation with temperature, **d** personalized three-time interval rheological sequence, which illustrates the thixotropic behavior of GEL40 and GEL4-CCM40 inks, **e** outcomes obtained in the drop test

Viscoelastic materials possess both an elastic modulus (or storage modulus, *G*′) and a viscous modulus (or loss modulus, *G*″) that rheology can dynamically quantify through a range of time and stresses, since these factors greatly impact the value of these moduli [34]. This analysis discerns if a material predominantly behaves as a solid or a fluid, which is an extremely useful pre-printing information, granting a better prediction of the printing outcomes and the possibility to promptly adapt specific printing parameters, such as temperature, to the formulation in use [18–20, 35]. For example, if a material shows a crossover between loss and storage moduli around 37 °C, it will not extrude adequately at 20 °C, as it will be excessively viscous (higher predominance of the elastic modulus), whereas a temperature of 60 °C will lead to an excessive predominance of the loss modulus and a sol-like status with inferior mechanical properties [19, 20]. Oscillatory temperature sweeps were performed to predict the ideal printing temperature window of the gelatin-based inks, which is approximately equal to the sol/gel transition temperature, identifiable through the crossover point (*G*′ = *G*″) [20, 36, 37], or through the point where the phase angle is equal to 45° [35, 38]. Analysis of Fig. 2a shows that, at 25 °C, the predominance of the elastic modulus and the resultant solid-like behavior is notorious and tends to be an order of magnitude higher than its viscous counterpart, for both GEL40 and GEL40-CCM40 inks. Oppositely, the discrepancy between moduli is almost intangible at 50 °C, with none of the moduli showing real predominance over the other, and overall, the material exhibiting fluid-like behavior. This phenomenon might not occur at higher temperatures, but temperatures above 50 °C held no interest due to the extensive harm they could cause to biological compounds [18].

The influence of CCM in the viscoelastic behavior of the hydrogel was examined by comparing the average sol/gel transition temperature of each formulation (Table 2 and Fig. 2b). For the control sample (GEL40), the crossover occurred at an average 41.4 ± 0.3 °C, whereas for GEL40-CCM40 (40% (w/w) medium), the average crossover temperature was of 38.7 ± 1.4 °C. These data suggest that the incorporation of CCM at this concentration does not affect the printing temperature. In terms of viscosity and complex modulus at the crossover (Table 2 and Fig. 2c), there was no significant difference in the mean values, suggesting that no further differences should be observed in terms of defining the ideal printing pressure.

**Table 2:** Rheological parameters (temperature, complex modulus, and viscosity) at the crossover point (sol–gel; *G*′ > *G″*)

**Formulations**	**Rheological parameters (mean ± SD, *n* = 3)**		
	**Sol–gel temperature (°C)**	G*^a^** at sol–gel (Pa)**	**Viscosity at sol–gel (*ɳ*, Pa·s)**
**GEL40**	41.4 ± 0.3	1090 ± 194	189.3 ± 18.7
**GEL40-CCM40**	38.7 ± 1.4	1052 ± 151	167.5 ± 37.8

^a^G* complex modulus

Another desirable feature of inks for bioprinting applications is thixotropy. Thixotropic materials experience a time-dependent recovery of viscosity at fixed shear rates, which is pivotal to obtain constructs with high shape fidelity [35]. After flowing through the nozzle walls and facing a decrease in viscosity, the ability to rapidly rearrange its internal structure in a resting state leads to a viscosity increase which hardens the ink, counteracts sagging forces, and provides enhanced printing quality [19, 39, 40]. To study the thixotropic behavior of the formulations, a three-time interval sequence was designed to mimic the different shear rates to which inks are subjected to during the printing process, with initial and final phases at low shear rates (to simulate the resting period inside the cartridge and the resting state after deposition, respectively) and a middle phase of high shear to mimic the printing extrusion process (Fig. 2d). The viscosity recovery behavior was obvious in the tested formulations when temperatures decreased. With relevance to the initial resting stage (constant shear rate of 0.1 s^−1^), inks experienced a viscosity increase until stabilization occurred. After the increment of the shear rate to 100 s^−1^, to simulate the stress exerted on the ink while extruding, all formulations had a large decrease in viscosity, as expected due to the shear-thinning behavior. Still, a slightly upwards tendency is visible across time, which may occur due to the rearrangement of polymer chains. Concerning the recovery phase, formulations had an effective viscosity recovery with a similar slope, with values starting to stabilize around 30 °C, meaning that the recovery behavior was identical over time. The GEL40-CCM40 had a recovery period similar to the other formulations, suggesting that the impact of CCM on printability is negligible, and that a decrease in printing quality is not expected when using CCM [35]. Viscosity levels in this phase were higher than at the initial resting phase due to the drop in temperature from 42 to 25 °C, highlighting the transition phase characteristic of the gelation process after ink extrusion. Therefore, these formulations can sustain the shape of filaments during and after printing.

To further validate the data obtained in the rheological analysis, a drop test was performed (Fig. 2e). This simple methodology is generally performed in the beginning of the optimization procedure to evaluate extrudability-related parameters. It consists in the static extrusion of material while the nozzle is suspended in the air, followed by the visual analysis of the printing outcome, to evaluate if the applied pressures are adequate to the gelation status of the ink [18, 41, 42]. However, even when applying the same set of parameters, these outcomes change with nozzle type and diameter. For instance, larger nozzle diameters require lower pressures. Typically, properly gelled inks create smooth hanging filaments, over-gelled inks lead to irregular and bumpy filaments, while droplets are formed when the inks are under-gelled [18, 23, 41]. Droplets can also be formed when insufficient pressures and low RT coincide, and the instant gelation of the inks occurs. Such effect might explain the lack of hanging filaments at 42 °C. Still, these outcomes did not invalidate the printing temperature interval defined in the rheology experiments.

#### Defining printing speed and flow rate

**Fig. 3:**
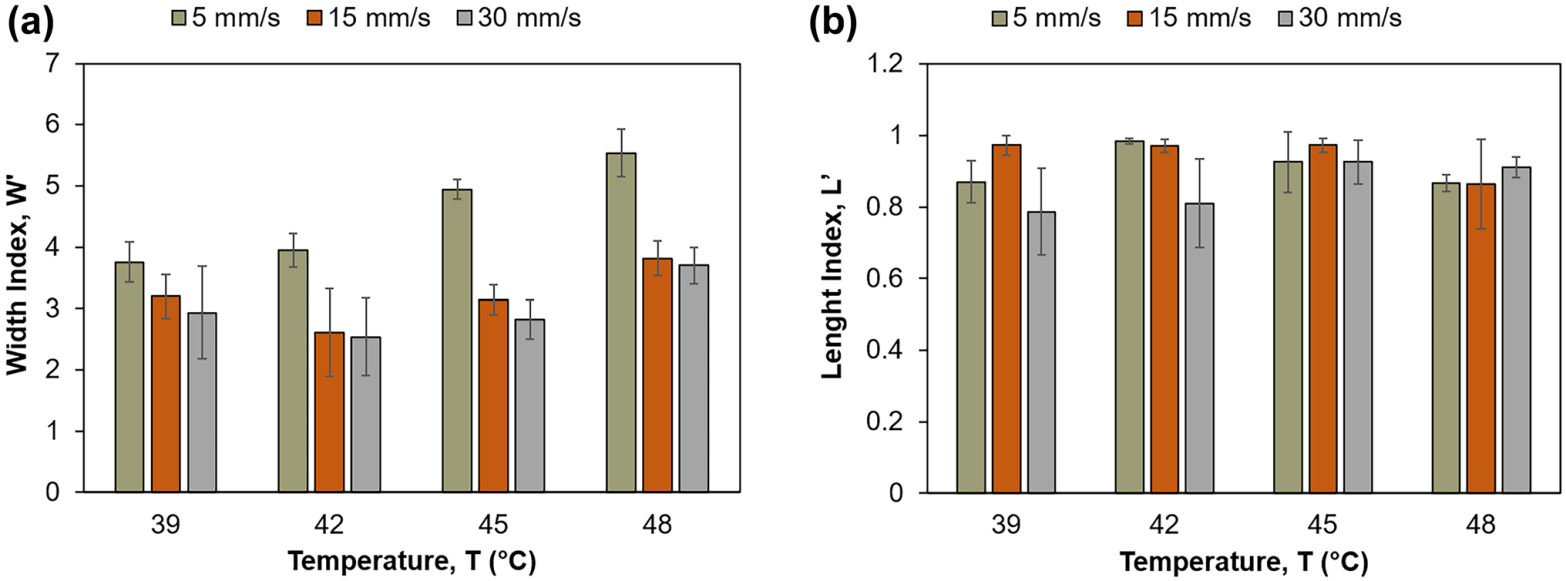
3D optimization outcomes at printing speed of 5, 15, and 30 mm/s. **a** Width index (mean ± SD), **b** length index (mean ± SD)

To further understand the impact of temperature selection on the printing accuracy, a line test was also performed. In a printing process, printing velocity and flow rate should be similar, meaning that when the pressure increases the printing speed should also increase, and vice versa. If this principle is not met, filaments will display either longitudinal or transversal elongation [19, 43, 44]. However, maintaining a constant extrusion flow in consecutive printing processes is difficult, since flow changes with several interrelated factors which can trigger a modification cascade after a single parameter is altered. For example, a slight increase in room temperature slows down the solidification process and promotes filament spreading [43, 45, 46]. Hence, the approach of printing linear filaments along a single axis at varying velocities is a common and simple methodology that allows to screen the relationship between temperature and pressure inputs with printing speed, which is generally accomplished by fitting filament measurements in indexes [19, 46, 47]. The average width index (*W*′) showed that 42 °C was the best temperature for printing within this range, as it rendered the lowest *W*′, which is consistent with the rheological characterization and illustrates the importance of the latter (Fig. 3a). There was also a tendency for *W*′ to increase with increasing temperatures, due to the ink being excessively liquid, which increased filament spreading. Regarding printing speeds, 5 mm/s was insufficient to accommodate the flow of extrusion yielding high *W*′. Oppositely, 15 and 30 mm/s speeds delivered much thinner filaments, depicting similar profiles across the measured temperatures. The average length index (*L*′) obtained shows that the metal tip did not achieve the perfect filament length, suggesting that it struggles to start extruding in the defined region, a fact which is further supported by the lack of filaments with an average *L*′ > 0.95 (i.e., over 38 mm in length) (Fig. 3b). Employing this range of velocities seemed to have had no significant effects on filament length, which agrees with results obtained by Kang et al. [26]. At this stage, and with the optimal temperature accurately defined, the pressure was adjusted to achieve an ideal flow rate. A flow rate of 2.5 µL/s was used in previous in-house experiments with remarkable printing quality being achieved; thus, the pressure was adjusted until that flow rate was achieved at 15 Psi.

#### Influence of angle direction and line distance on printing accuracy and pore area

**Fig. 4:**
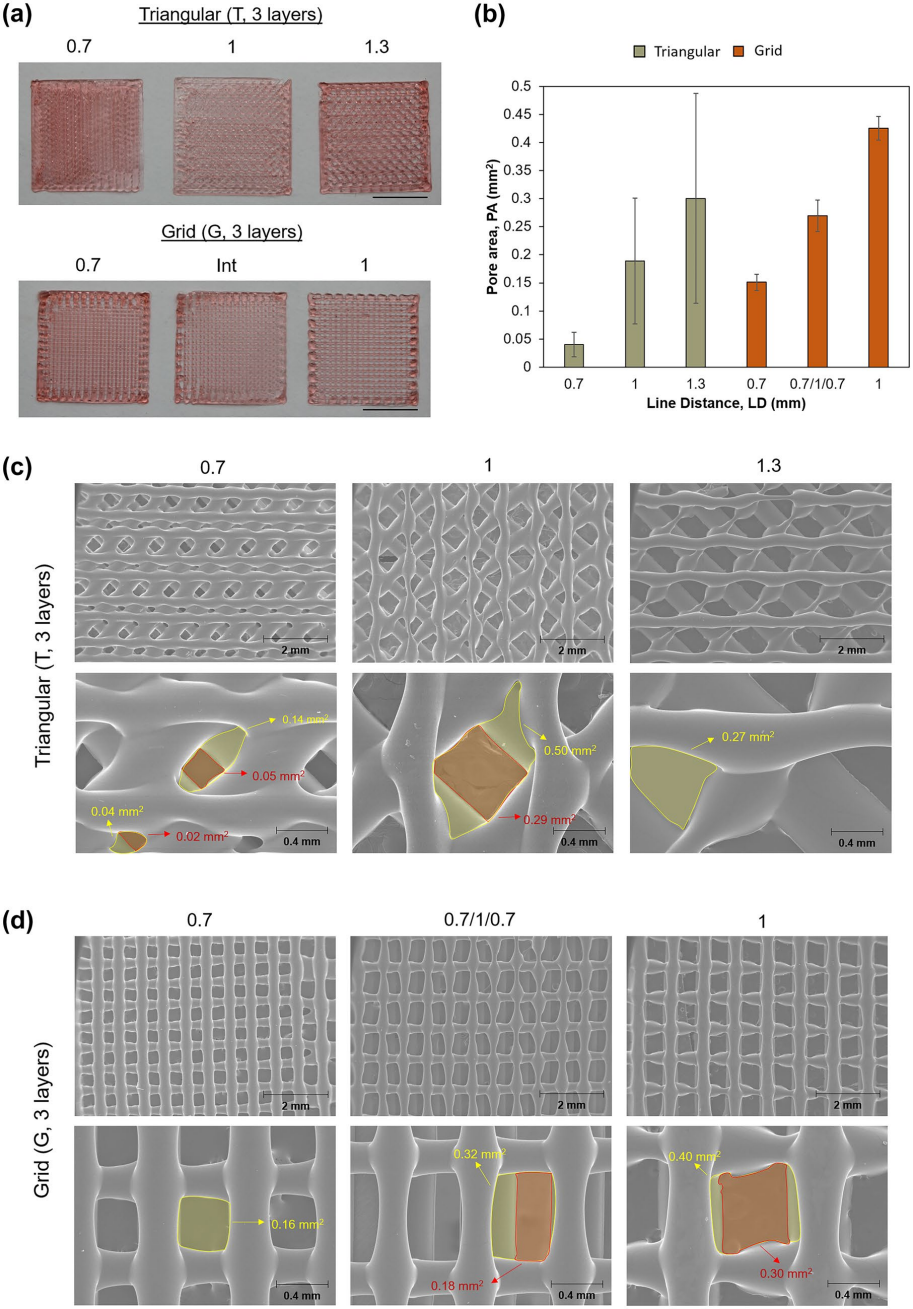
Gelatin-based patches printed at two different infill patterns (T and G) with varying spacing between filaments (line distance, LD). **a** Macroscopic observations of the triangular (T) and grid (G) printed structures; scale bar = 10 mm, **b** pore area measurement presented as mean ± SD (*n* = 10), **c** scanning electron microscopy (SEM) images of T patches highlighting the differences in pore area, PA (i.e., within the same patch—impact of layer’s height, and between patches), **d** scanning electron microscopy (SEM) images of G patches highlighting the differences in pore area, PA (i.e., within the same patch—impact of layer’s height, and between patches)

The design characteristics of 3D-printed dressings are a pivotal feature when determining their applicability, with no single design excelling in all types of skin lesions [13]. For instance, a smaller distance between adjacent filaments will lead to compact and denser vehicles, enhancing their compressive modulus [17] and slowing down release, whereas the orientation of subjacent filaments will influence their tensile strength [48]. Currently, an ideal design can be achieved using a quality by design (QbD) approach, making the process costly and time-consuming [27, 49, 50]. It is, therefore, fundamental to understand the implications of modifying such design parameters to rapidly adjust to the researchers’ needs. To achieve this, dressings were printed with different filament spacing and orientations, and the mean pore areas (Fig. 4a and b) and release profiles were analyzed.

The average pore area in dressings with the grid format (G) was associated with a much smaller standard deviation than in triangular patterns (T), meaning that pore size is more consistent in grid patches throughout the whole pool. This can be explained by skewed filaments originating both larger and smaller diamond-shaped pores when a new layer is deposited, which disrupts the mean value. Another phenomenon more preeminent in the triangular deposition was the disturbance of the void area along the z-axis, which can lead to different interpretations of pore size (Fig. 4c). Theoretically, this happens less in grid-like dressings due to the perfect overlaying of intercalated filaments but, in practice, defects in printing accuracy will also form pores with distinct heights (Fig. 4d).

Overall, the T-0.7 patches were the most consistent and with the smallest pore area, with an average value of 0.04 ± 0.02 mm^2^. Therefore, it is expected that this smaller macroporosity level depicts a much more controlled release than its grid counterpart [50]. According to Milojevic et al. [13], this mean pore area nears the recommended value for skin wound dressings as it promotes an adequate ingrowth of tissue and capillaries. Concomitantly, this filament spacing seemed to minimize the variability of pore area, delivering pores with a much more consistent size relative to the rest of the T pool. Indeed, T-1 (PA = 0.19 ± 0.11 mm^2^) and T-1.3 (PA = 0.30 ± 0.19 mm^2^) had significantly larger errors associated, with an increase in the SD of around fivefold for each increment of 0.3 mm made in filament spacing. This discrepancy in pore area may, however, represent an advantage over dressings with further pore definition, which can be used for applications that involve cell incorporation [13].

Oppositely, the average pore area on grid layouts was much more consistent in the whole pool. The G-0–7 (PA = 0.15 ± 0.01 mm^2^) patch seemed the most accurately printed construct, as evidenced by the low SD. Comparatively, G-1 (PA = 0.43 ± 0.02 mm^2^) looks to have endured some sagging in the second layer, which slightly disturbed the pore contour. Moreover, it was shown that increasing filament spacing by 0.3 mm with this design leads to an increase of around threefold in pore area, which is consistent with the data reported by Tytgat et al. [17] in similar grid constructs. Patches with 1.3-mm filament spacing were printed (data no shown) but discarded due to large pore size, which led to an excessive release rate. For this reason, we intercalated a middle layer with a 1-mm spacing between two layers of 0.7 mm (G-0.7/1/0.7), to study the effects of combining different values of filament spacing. G-3 had an average pore area of 0.27 ± 0.03 mm^2^, which is almost the double of G-0.7. This range of pore area was observed by Tytgat et al. [17], in patches with a filament spacing of 0.8 mm, which highlights the importance of this type of research by emphasizing that it is possible to achieve similar pore-related features when utilizing distinct designs, possibly overcoming design-related incompatibilities that can sometimes hinder the application of 3D-printed medical devices. Moreover, G-0.7/1/0.7 had a noticeable higher SD which might be due the elongation of the uppermost layer or to the excessive spreading between base layer and support material, which also seems to cause unaligned layers that spoil a uniform pore area along the entirety of the z-axis. On the other hand, this misalignment can lead to an increase in pore interconnectivity due to more gaps being opened between neighboring pores [51].

### In vitro release for different porous patches geometries

To understand and quantify how the release profile and kinetics are influenced by the pore area and design geometry of the CCM gelatin-based vehicles, the probe rhodamine B (RB, model bioactive) and bovine serum albumin (BSA, model protein) were used in different patches selected from the previous assays, namely, G-0.7/1/0.7, T-0.7, and T-1.3. A patch with 100% infill was used as control.

**Fig. 5:**
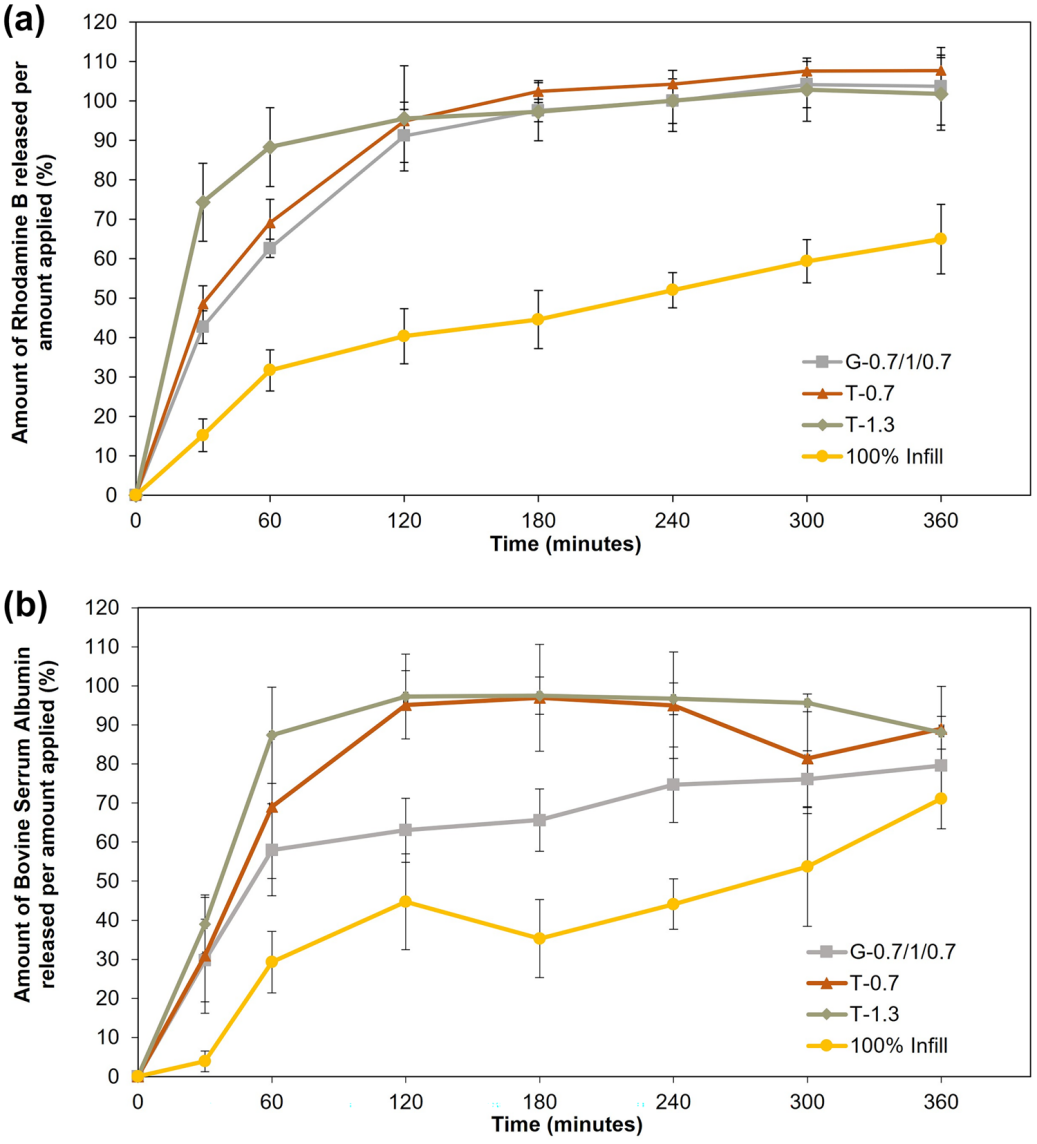
Release profiles of rhodamine B (**a**) and bovine serum albumin (**b**) from occlusive (100% infill) and non-occlusive (G-0.7/1/0.7, T-1.3, and T-0.7) patches printed with GEL40-CCM40 inks (mean ± SD; *n* = 6) G-0.7/1/0.7, 3-layered grid with a line distance of 0.7–1-0.7, T-1.3, 3-layered triangular with a line distance of 1.3, T-0.7, 3-layered triangular with a line distance of 0.7

As shown in Fig. 5, the release of RB and BSA from the matrices of the porous patches was faster than that observed from the occlusive, 100% infill control patch. No significant differences were observed between RB-containing triangular and grid filling, but the distance between lines influenced the early RB release, with T-1.3 releasing 74.36% of the probe within the first 30 min and T-0.7 only releasing 48.56% during the same period. Interestingly, the G-0.7/1/0.7 patch (PA = 0.27 ± 0.03 mm^2^), despite having a mean pore area around seven times higher than that of T-0.7 (PA = 0.04 ± 0.02 mm^2^), showed a similar release profile, a result which is worthwhile exploring in future studies. For porous and grid patches with BSA, an identical release profile was observed, although differences in the release rate can be distinguished at each time point (mean value). Furthermore, this data suggests that for BSA release, differences can be portrayed between triangular and grid patches, with the release rate increasing according to the following order: G-0.7/1/0.7/T-0.7/T-1.3. This data agrees with the patches’ porosity area differences, although the influence of the network topology can also be observed, as discussed for RB patches. Moreover, the T-1.3 patch shows a strong correlation between RB and BSA release—similar profile.

Overall, after 6 h all the RB-patches had released the total RB content, except the control patch, which only released 65.04% of the probe. For BSA patches, the control patch released a total of 71.07%, whereas the G-0.7/1/0.7, T-0.7, and T-1.3 released 79.56%, 89.01%, and 88.01%, respectively. Thus, occlusive patches (100% infill) are probably more adequate for sustained and prolonged release. Release from non-occlusive patches was much faster at early time points, which may be due to either fast diffusion or fast degradation of the patch in the beginning of the experiment (burst release), which gradually slows down. However, the differences observed in the release rates from the non-occlusive patches must be carefully evaluated as they represent a great opportunity for customizing such rates by changing the filament spacing.

There are several in vitro methods which describe the overall release from drug-loaded vehicles, allowing a better prediction of the in vivo performance [52, 53]. In general, a biodegradable polymeric matrix will release its content due to a diffusion, erosion, or degradation mechanism [54–56]. Therefore, to define the release profile of our vehicle, well-known release kinetics models were employed, namely, the zero order, first order, Higuchi, Korsmeyer-Peppas, and Weibull models. The parameters obtained with these models are summarized in Table S1 (Supplementary material).

The fitting of the zero-order model to the release data obtained for all patches was poor, as revealed by the low *R*^2^_adjusted_ obtained. This is not surprising, since this model works best for loaded vehicles which do not degrade (which is not a characteristic of gelatin matrices) and for slow, constant, concentration-independent drug release; zero-order kinetics is only a function of time [53, 55, 57]. Concerning the first-order kinetic model, the adjusted *R*^2^_adjusted_ suggests a generally good fit only for RB-containing patches. First-order equations describe a system where the concentration of drug being released over time is concentration-dependent [57]. For porous matrices, the amount of active agent released is proportional to the amount remaining in the carrier, therefore tending to decrease over time [57]. The Higuchi model was the first to describe the release of active agents from matrix systems, initially conceived for planar systems. Nevertheless, it was expanded to accommodate different geometries and porous matrices, either solid or semi-solid, and for drugs with both low and high solubility [53, 55, 57]. It is adequate for a myriad of applications aiming to describe dissolution in several dosage forms, including matrix-based transdermal systems, as is the case in this application. The fitness of this model was only satisfactory for RB-occlusive (*R*^2^_adjusted_ = 0.967 ± 0.026), RB-G-0.7/1/0.7 (*R*^2^_adjusted_ = 0.872 ± 0.068), and RB-T-0.7 (*R*^2^_adjusted_ = 0.850 ± 0.055) patches. However, for the RB- and BSA-T-1.3 (patches with the largest pores) and BSA-occlusive, BSA-T-0.7, and BSA-G-0.7/1/0.7, the fitting was not adequate, probably due to this model’s assumption that the swelling and dissolution of the matrix is negligible. Overall, this data suggest that this model cannot be applied to the triangular and grid-like gelatin-based patches, because their fragmentation was clear during the release test; this model do not mimic their dissolution profile.. The Korsmeyer-Peppas model also describes drug release from polymeric systems [52, 53], and fitting of this model was also good, as shown by the obtained *R*^2^_adjusted_ values. The *n* value, although more adapted to cylindrical shaped matrices [52, 53], suggests that Fickian diffusion occurs in all patches, since *n* ~ 0.5 [57]. The Weibull model, adapted to describe different dissolution processes [53], is also useful for the comparison of drug release profiles from matrix systems [53, 57]. Indeed, there was a relatively good fitting for this model with overall *R*^2^_adjusted_ above 0.968 for RB patches; for BSA patches the fitting was acceptable only for the T-0.7 patch (*R*^2^_adjusted_ = 0.865). The alpha (*α*) parameter, which is utilized to describe the time-dependence of the release process [57], showed higher values for RB-containing occlusive, G-0.7/1/0.7, and T-0.7 patches, thus indicating that dissolution and release occurred for a longer period than for the T-1.3 patch, corroborating the cumulative release results. Interestingly, and considering the BSA release, occlusive and T-1.3 patches showed much higher values than those obtained for T-0.7 and G-0.7/1/0.7 patches, which the differences in the release profile can explain. The beta (*β*) parameter describes the shape of the dissolution curve; for *β* < 1, the dissolution curves show a high initial slope [57]. RB-occlusive, RB-G-0.7/1/0.7, RB-T-0.7, BSA-T-0.7, and BSA-G-0.7/1/0.7 patches had *β* values higher that obtained for the RB-T-1.3, BSA-T-1.3, and BSA-occlusive patch, which means that the dissolution profile was less steep, again supporting a slower drug release for the first 5 patches.

## Conclusions

The mechanisms leading to skin inflammatory diseases remain incompletely understood and the existing treatments are usually poorly effective and/or have serious side effects; thus, there is a need to develop topical personalized therapies using state-of-the-art technology. The use of 3D printing for pharmaceutical applications has been increasing, aiming to develop innovative, cost-effective, sustainable, and personalized treatment platforms [58]. It was, therefore, of interest, to study a practical approach to readily create such vehicles, which feature the desired pore area and network topology for local, topical, and personalized call-based bioactives applications.

Overall, this study showed that the incorporation of CCM in the ink formulation did not impair the quality of the constructs, which is of extreme importance to translational purposes. Specifically, rheological analysis showed that CCM hydrogels had crossover temperatures closer to the physiological range (~ 37 °C). Furthermore, the 3D printing data showed that, by varying the printing settings, it is possible to produce CCM-based hydrogel patches with different degrees of porosity, which greatly impact the release profile, thus reinforcing the pertinence of using such technologies to design personalized topical patches with desired features for different skin applications. The results also showed that the pore area increased with increasing filament spacing for both grid and triangular infills. In addition, the triangular infill was the design which rendered proportionally smaller average pore areas at each spacing tested, albeit with much larger standard deviations, which indicates a wider range of pore sizes. On the other hand, grid layouts had increased printing accuracy and delivered better pore definition. The release results revealed that larger filament spacing led to faster release, as expected. Interestingly, the RB- and BSA-containing G-0.7/1/0.7 patches, despite having around a seven-fold higher mean pore area than T-0.7, showed a similar release profile, which can be linked to the network topology of the printed structures. Further studies are needed to characterize the same or different materials in other designs, like honeycomb and circular meshes, and to assess the influence of different number of layers, for instance. This will lead to a better understanding of the relationship between design-dependent pore areas and the consequent release rate and mechanisms of the dressing, giving insight into how to adapt the patch design to the desirable rate of controlled bioactive release in a fast manner.

In conclusion, this work delivered insight over the practicality of employing (bio)printing in the production of carriers with reproducible and controlled pore area for topical drug delivery, amenable to the incorporation of cell-derived secretome. Such scenario offers a great versatility to this kind of vehicle which can be quickly modified to the different requirements of different skin lesions and diseases. Furthermore, relapsing skin diseases are remarkably suited to this technology as the imaging of skin lesions can be readily transposed into personalized patches, whose designs can be then stored in a database, and which can be promptly re-printed when subsequent relapsing events arise.

## Supplementary Information

Below is the link to the electronic supplementary material.

Supplementary file1 (DOCX 26 KB)

## Data Availability

Not applicable.
