# Supplementary material for: Towards the personalization of gelatin-based 3D patches: a tunable porous carrier for topical applications
Source: Drug Deliv Transl Res. 2023 Jan 12;13(6):1799–812. doi: 10.1007/s13346-023-01294-y (PMC10125939; doi:10.1007/s13346-023-01294-y)
Supplement: Supplementary file 1 — Supplementary file1 (DOCX 26 KB) [file 13346_2023_1294_MOESM1_ESM.docx]

**Towards the personalization of gelatin-based 3D patches: A tunable porous carrier for topical applications**

**Ricardo Ribeiro ^a^, Sara Bom ^a^, Ana M. Martins ^a^, Helena M. Ribeiro ^a^, Catarina Santos ^a,b,c^, Joana Marto ^a^***

^a^ Research Institute for Medicines (iMed.ULisboa), Universidade de Lisboa, 1649-003 Lisboa, Portugal; sarabom@campus.ul.pt (S.B.); ris.ribeiro@campus.fct.unl.pt (R.R.); amartins@farm-id.pt (A.M.M.); hribeiro@campus.ul.pt (H.M.R.); catarina.santos@estsetubal.ips.pt (C.S.); jmmarto@ff.ulisboa.pt (J.M.).

^b^ CQE, Instituto Superior Técnico, Universidade de Lisboa, Av. Rovisco Pais 1049-001, Lisboa, Portugal.

^c^ EST Setúbal, CDP2T, Instituto Politécnico de Setúbal, Portugal.

***** Correspondence: jmmarto@ff.ulisboa.pt.

**SUPPLEMENTARY MATERIAL**

**Table S1** Parameters obtained by fitting 5 different kinetic models to the release data from the RB 3D patches (mean ± SD; n=6)

| Infill | Model | k | R^2^_adjusted_ | AIC |
| --- | --- | --- | --- | --- |
| G-0.7/1/0.7 | Zero order | 0.384 ± 0.021 | 0.311 ± 0.130 | 72.578 ± 1.158 |
|  | First order | 0.018 ± 0.002 | 0.976 ± 0.013 | 44.748 ± 5.435 |
|  | Higuchi | 6.437 ± 0.316 | 0.872 ± 0.068 | 58.350 ± 3.762 |
|  | Korsmeyer-Peppas | 10.567 ± 3.245  n = 0.426 ± 0.086 | 0.857 ± 0.044 | 60.460 ± 3.354 |
|  | Weibull | α = 44.624 ± 14.036  β = 0.933 ± 0.102 | 0.969 ± 0.019 | 47.146 ± 6.074 |
| T-0.7 | Zero order | 0.400 ± 0.010 | 0.220 ± 0.144 | 78.869 ± 1.467 |
|  | First order | 0.026 ± 0.004 | 0.963 ± 0.025 | 47.997 ± 5.560 |
|  | Higuchi | 6.775 ± 0.185 | 0.850 ± 0.055 | 60.388 ± 2.937 |
|  | Korsmeyer-Peppas | 10.694 ± 2.035  n = 0.410 ± 0.062 | 0.326 ± 0.738 | 69.375 ± 9.640 |
|  | Weibull | α = 83.314 ± 41.557  β = 1.132 ± 0.093 | 0.974 ± 0.012 | 46.968 ± 4.072 |
| T-1.3 | Zero order | 0.470 ± 0.187 | -0.359 ± 0.409 | 63.784 ± 15.530 |
|  | First order | 0.043 ± 0.034 | 0.886 ± 0.120 | 44.519 ± 10.131 |
|  | Higuchi | 7.110 ± 0.876 | 0.562 ± 0.169 | 56.135 ± 14.500 |
|  | Korsmeyer-Peppas | 47.634 ± 29.059  n = 0.160 ± 0.128 | 0.943 ± 0.079 | 41.426 ± 11.170 |
|  | Weibull | α = 3.717 ± 3.192  β = 0.373 ± 0.179 | 0.970 ± 0.034 | 40.661 ± 8.968 |
| 100% Infill | Zero order | 0.209 ± 0.022 | 0.740 ± 0.096 | 48.700 ± 10.145 |
|  | First order | 0.003 ± 0.001 | 0.891 ± 0.058 | 42.242 ± 10.050 |
|  | Higuchi | 3.421 ± 0.387 | 0.967 ± 0.026 | 32.380 ± 12.411 |
|  | Korsmeyer-Peppas | 3.679 ± 2.225  n = 0.514 ± 0.112 | 0.962 ± 0.033 | 33.587 ± 13.277 |
|  | Weibull | α = 59.878 ± 35.071  β = 0.671 ± 0.120 | 0.968 ± 0.032 | 32.332 ± 12.893 |

**Table S2** Parameters obtained by fitting 5 different kinetic models to the release data from the BSA 3D patches (mean ± SD; n=6)

| Infill | Model | k | R^2^_adjusted_ | AIC |
| --- | --- | --- | --- | --- |
| G-0.7/1/0.7 | Zero order | 0.314 ± 0.062 | -3.980 ± 5.016 | 37.636 ± 19.515 |
|  | First order | 0.008 ± 0.002 | -1.413 ± 2.955 | 34.044 ± 15.945 |
|  | Higuchi | 4.826 ± 0.149 | -0.258 ± 1.213 | 32.793 ± 16.456 |
|  | Korsmeyer-Peppas | 16.227 ± 8.730  n = 0.340 ± 0.265 | 0.688 ± 0.161 | 28.516 ± 18.830 |
|  | Weibull | α = 9.055 ± 9.077  β = 0.362 ± 0.242 | 0.507 ± 0.243 | 41.339 ± 6.787 |
| T-0.7 | Zero order | 0.309 ± 0.049 | -4.486 ± 7.420 | 51.852 ± 5.826 |
|  | First order | 0.015 ± 0.010 | 0.553 ± 0.384 | 39.840 ± 12.055 |
|  | Higuchi | 5.361 ± 0.900 | -0.790 ± 2.250 | 45.984 ± 5.379 |
|  | Korsmeyer-Peppas | 24.618 ± 17.284  n = 0.246 ± 0.080 | 0.427 ± 0.096 | 43.036 ± 9.238 |
|  | Weibull | α = 8.393 ± 15.726  β = 0.403 ± 0.497 | 0.865 ± 0.090 | 33.403 ± 5.345 |
| T-1.3 | Zero order | 0.374 ± 0.041 | -2.027 ± 1.569 | 52.857 ± 13.581 |
|  | First order | 0.022 ± 0.005 | 0.846 ± 0.110 | 35.294 ± 11.100 |
|  | Higuchi | 6.288 ± 0.333 | 0.560 ± 0.028 | 46.052 ± 13.075 |
|  | Korsmeyer-Peppas | 26.965 ± 9.333  n = 0.350 ± 0.239 | 0.504 ± 0.247 | 43.041 ± 12.255 |
|  | Weibull | α = 1065.999 ± 1248.653  β = 1.288 ± 0.762 | 0.679 ± 0.225 | 43.260 ± 13.019 |
| 100% Infill | Zero order | 0.200 ± 0.011 | 0.412 ± 0.390 | 46.190 ± 5.751 |
|  | First order | 0.003 ± 0.001 | 0.555 ± 0.260 | 45.393 ± 3.799 |
|  | Higuchi | 3.215 ± 0.214 | 0.638 ± 0.134 | 44.882 ± 3.090 |
|  | Korsmeyer-Peppas | 2.903 ± 2.619  n = 0.617 ± 0.247 | 0.623 ± 0.226 | 45.040 ± 3.284 |
|  | Weibull | α = 311.922 ± 379.636  β = 0.804 ± 0.320 | 0.617 ± 0.202 | 45.434 ± 2.818 |
